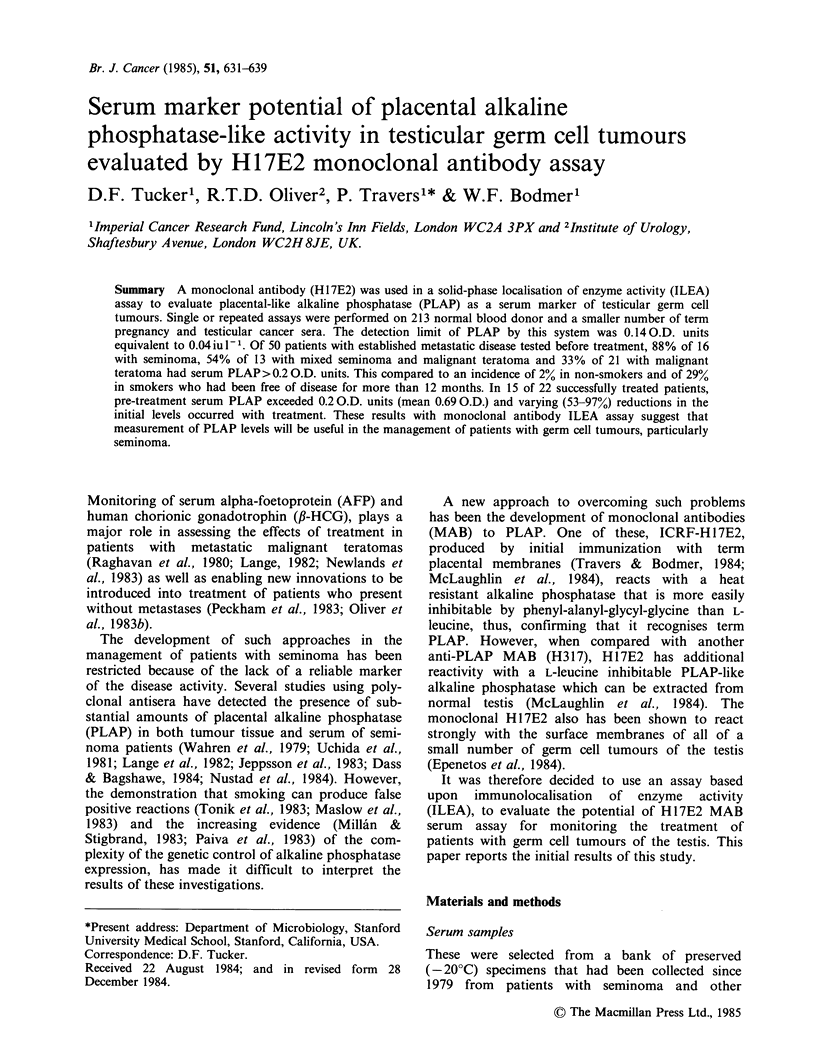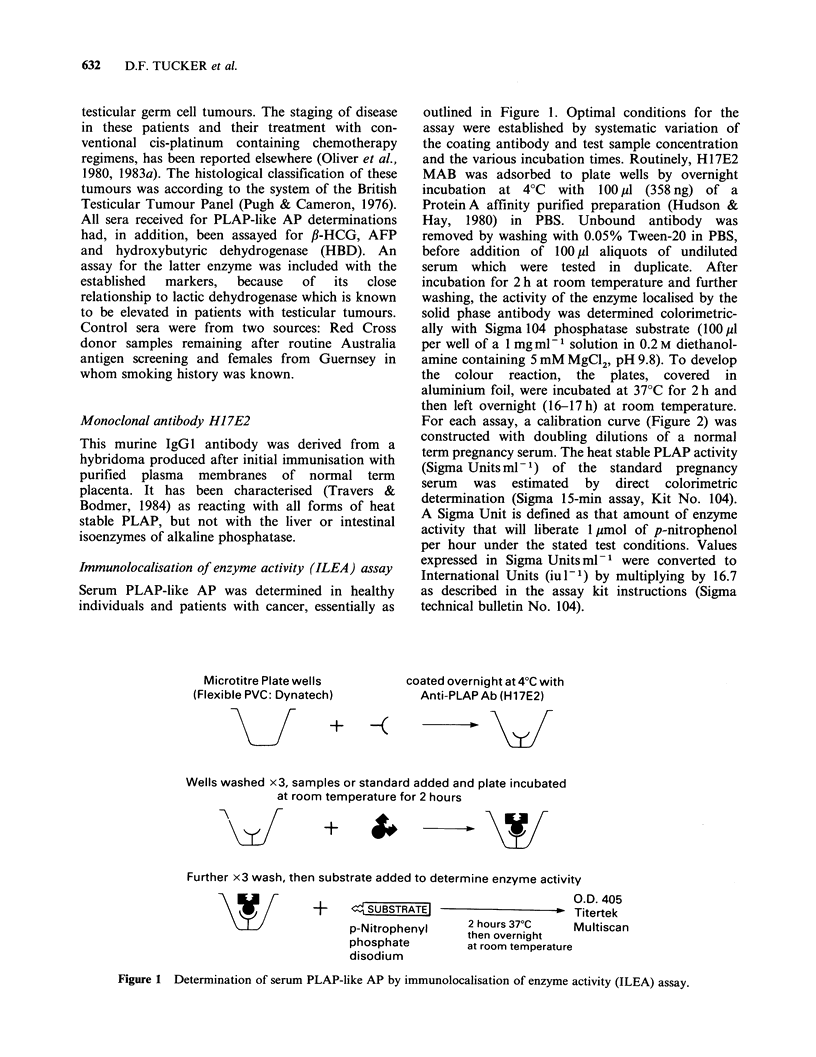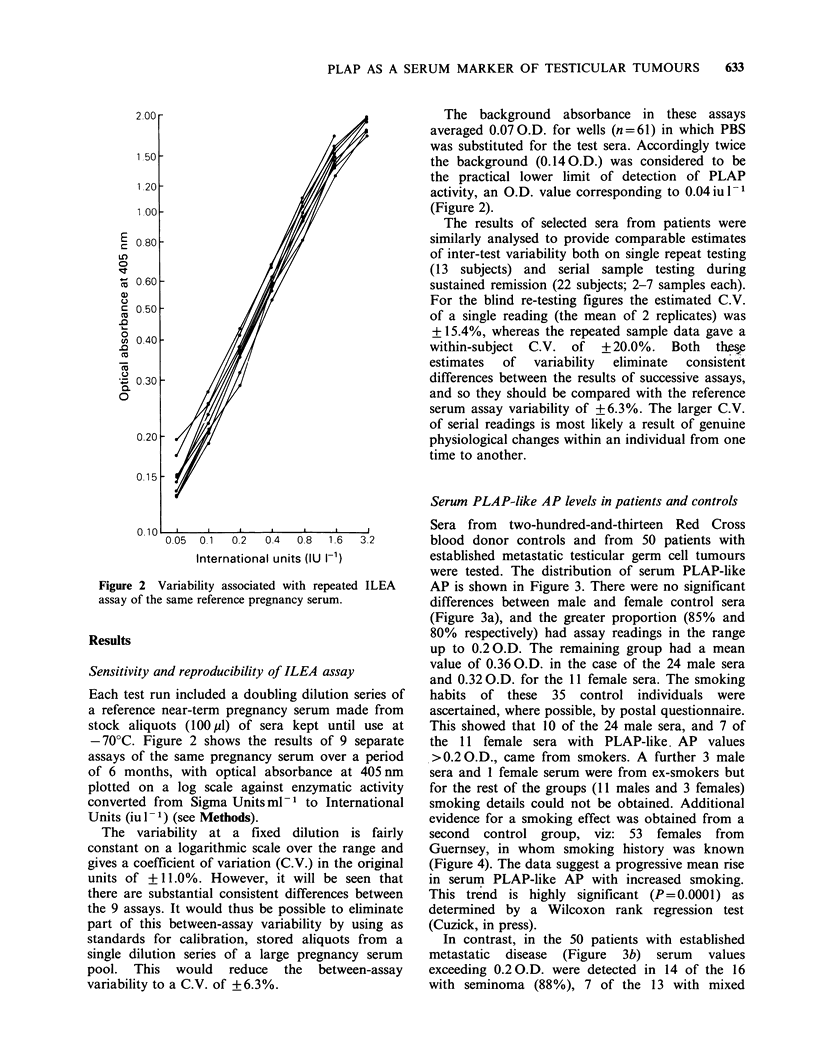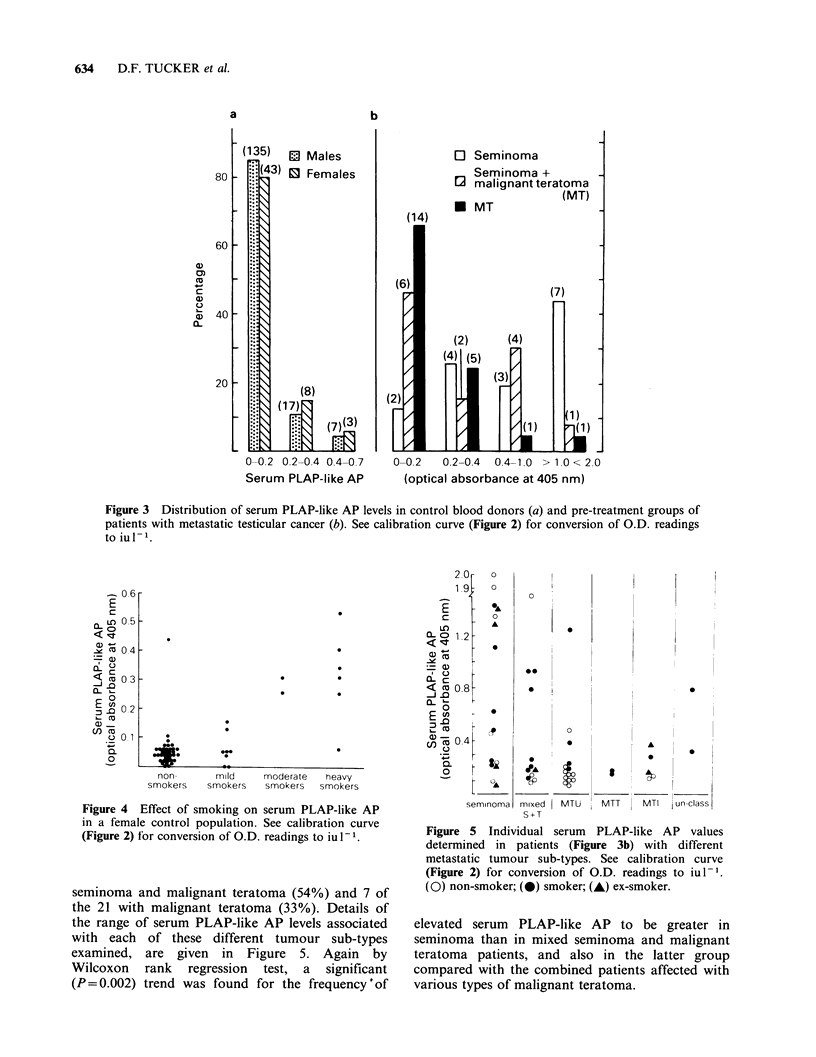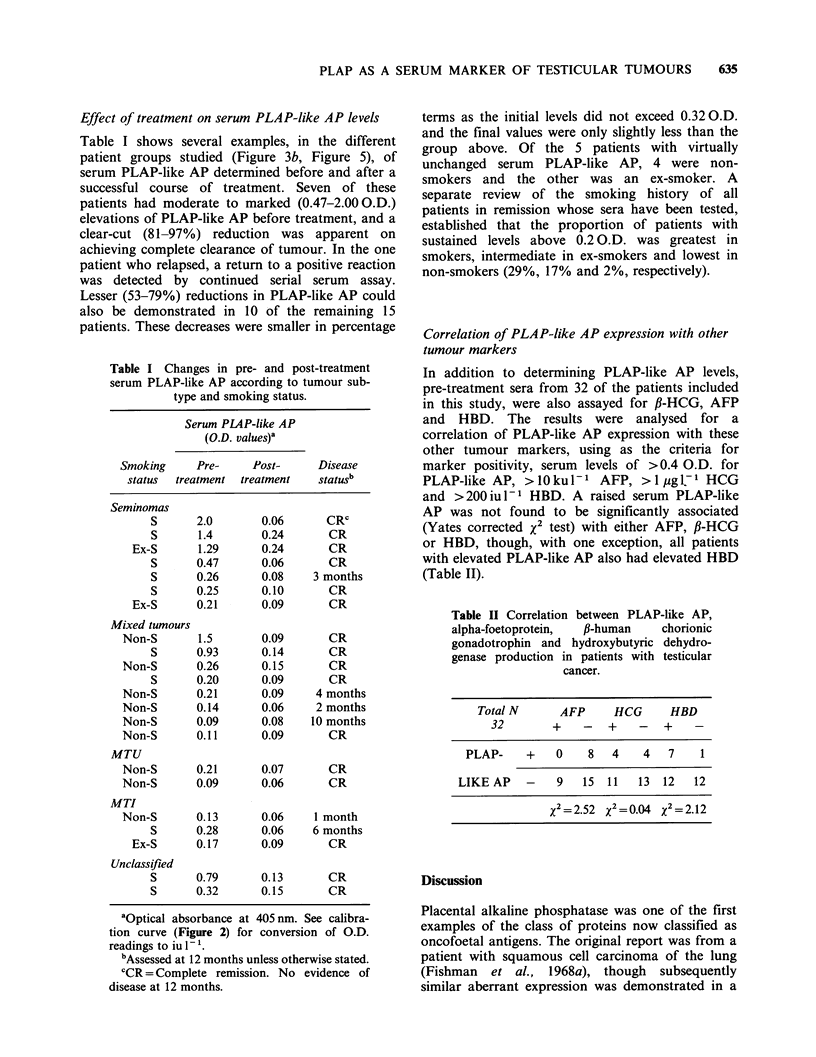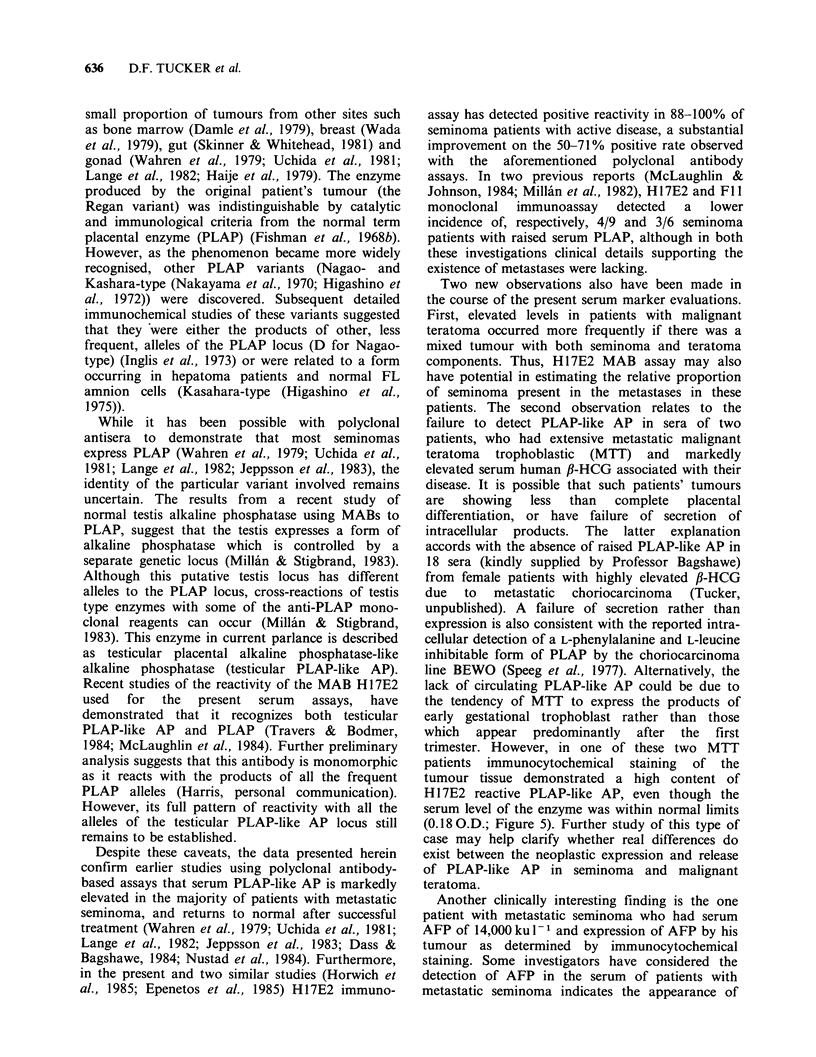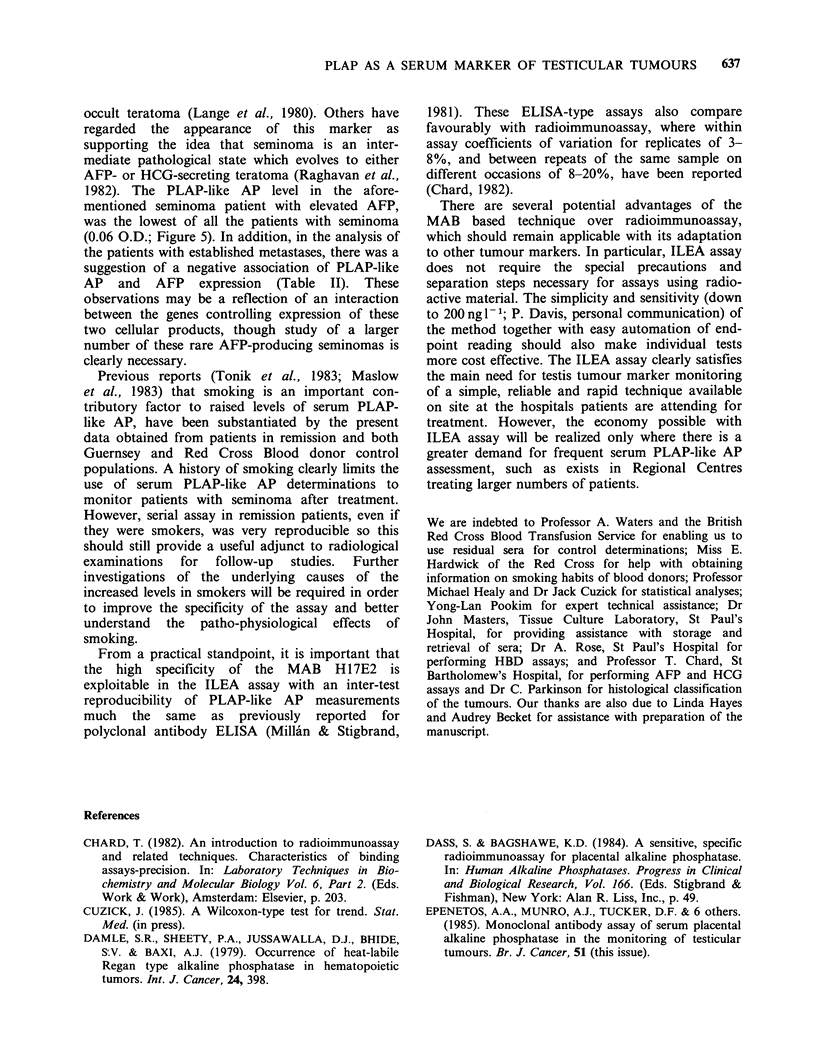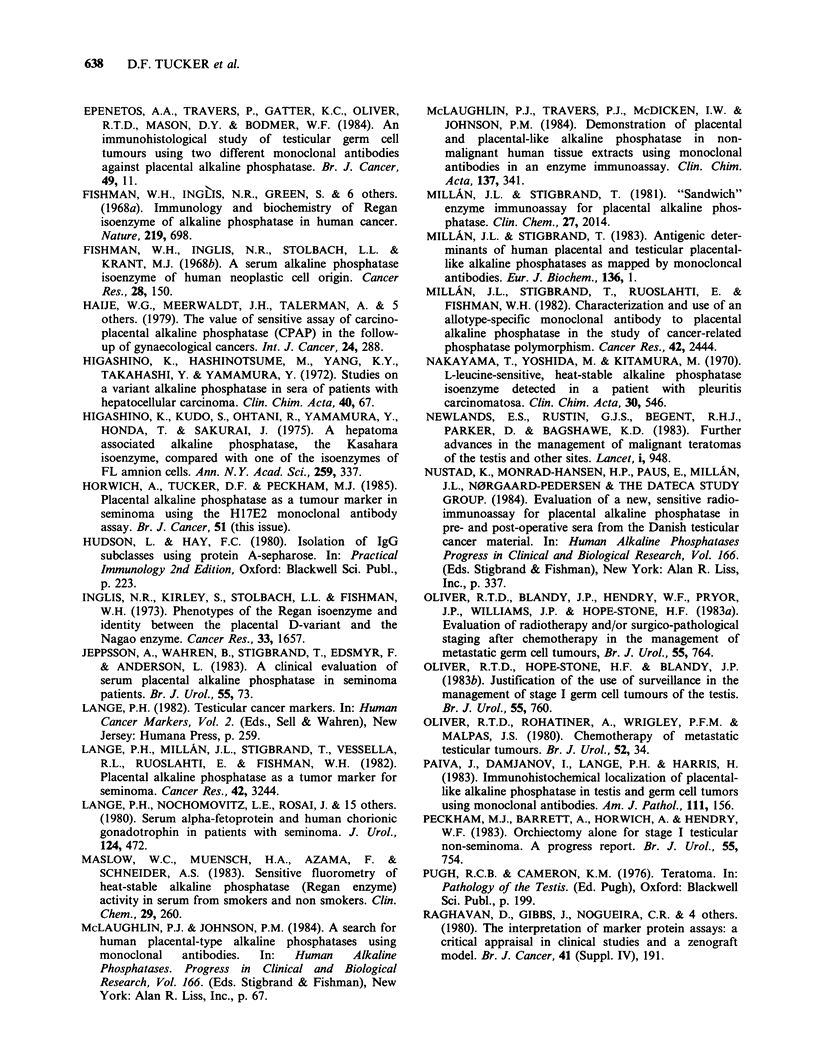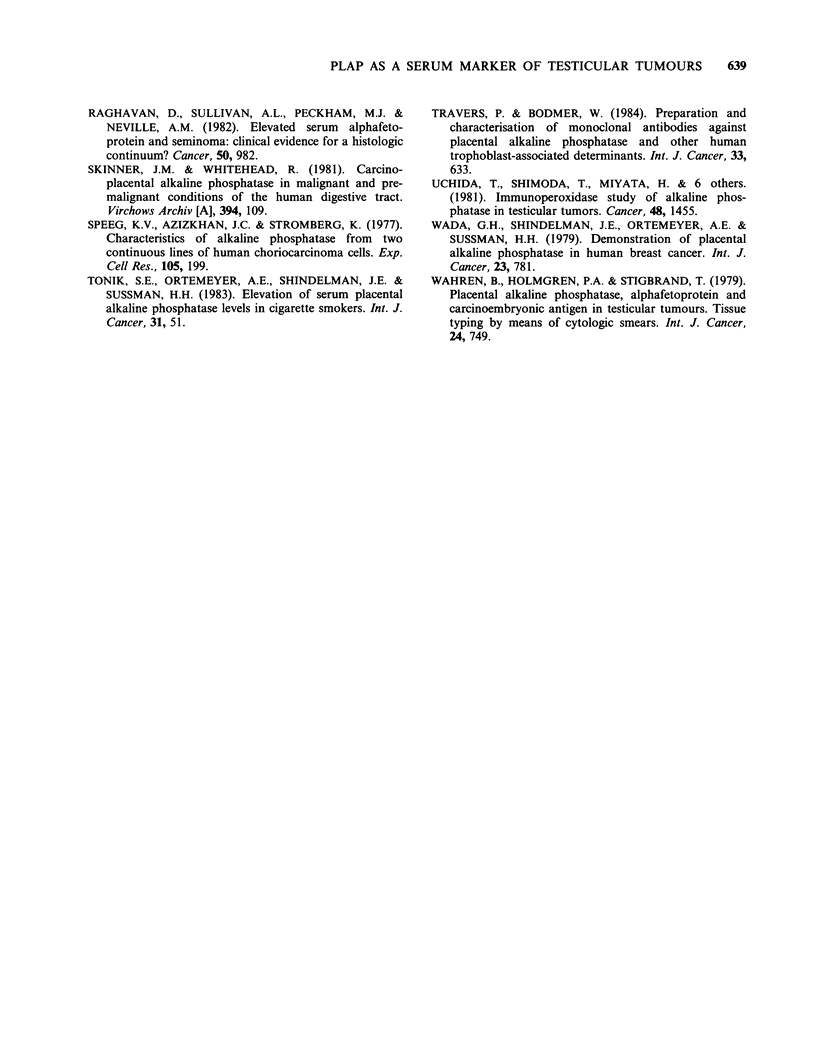# Serum marker potential of placental alkaline phosphatase-like activity in testicular germ cell tumours evaluated by H17E2 monoclonal antibody assay.

**DOI:** 10.1038/bjc.1985.95

**Published:** 1985-05

**Authors:** D. F. Tucker, R. T. Oliver, P. Travers, W. F. Bodmer

## Abstract

A monoclonal antibody (H17E2) was used in a solid-phase localisation of enzyme activity (ILEA) assay to evaluate placental-like alkaline phosphatase (PLAP) as a serum marker of testicular germ cell tumours. Single or repeated assays were performed on 213 normal blood donor and a smaller number of term pregnancy and testicular cancer sera. The detection limit of PLAP by this system was 0.14 O.D. units equivalent to 0.04iul-1. Of 50 patients with established metastatic disease tested before treatment, 88% of 16 with seminoma, 54% of 13 with mixed seminoma and malignant teratoma and 33% of 21 with malignant teratoma had serum PLAP greater than 0.2 O.D. units. This compared to an incidence of 2% in non-smokers and of 29% in smokers who had been free of disease for more than 12 months. In 15 of 22 successfully treated patients, pre-treatment serum PLAP exceeded 0.2 O.D. units (mean 0.69 O.D.) and varying (53-97%) reductions in the initial levels occurred with treatment. These results with monoclonal antibody ILEA assay suggest that measurement of PLAP levels will be useful in the management of patients with germ cell tumours, particularly seminoma.


Br. J. Cancer (1985), **51**, 631–639

### Serum marker potential of placental alkaline phosphatase-like activity in testicular germ cell tumours evaluated by H17E2 monoclonal antibody assay

D.F. Tucker[1], R.T.D. Oliver[2], P. Travers[1]* & W.F. Bodmer[1]

[1]*Imperial Cancer Research Fund, Lincoln's Inn Fields, London WC2A 3PX and* [2]*Institute of Urology, Shaftesbury Avenue, London WC2H 8JE, UK.*

**Summary** A monoclonal antibody (H17E2) was used in a solid-phase localisation of enzyme activity (ILEA) assay to evaluate placental-like alkaline phosphatase (PLAP) as a serum marker of testicular germ cell tumours. Single or repeated assays were performed on 213 normal blood donor and a smaller number of term pregnancy and testicular cancer sera. The detection limit of PLAP by this system was 0.14 O.D. units equivalent to $0.04\,\mathrm{iu\,l^{-1}}$. Of 50 patients with established metastatic disease tested before treatment, 88% of 16 with seminoma, 54% of 13 with mixed seminoma and malignant teratoma and 33% of 21 with malignant teratoma had serum PLAP > 0.2 O.D. units. This compared to an incidence of 2% in non-smokers and of 29% in smokers who had been free of disease for more than 12 months. In 15 of 22 successfully treated patients, pre-treatment serum PLAP exceeded 0.2 O.D. units (mean 0.69 O.D.) and varying (53–97%) reductions in the initial levels occurred with treatment. These results with monoclonal antibody ILEA assay suggest that measurement of PLAP levels will be useful in the management of patients with germ cell tumours, particularly seminoma.

Monitoring of serum alpha-foetoprotein (AFP) and human chorionic gonadotrophin ($\beta$-HCG), plays a major role in assessing the effects of treatment in patients with metastatic malignant teratomas (Raghavan *et al.*, 1980; Lange, 1982; Newlands *et al.*, 1983) as well as enabling new innovations to be introduced into treatment of patients who present without metastases (Peckham *et al.*, 1983; Oliver *et al.*, 1983*b*).

The development of such approaches in the management of patients with seminoma has been restricted because of the lack of a reliable marker of the disease activity. Several studies using polyclonal antisera have detected the presence of substantial amounts of placental alkaline phosphatase (PLAP) in both tumour tissue and serum of seminoma patients (Wahren *et al.*, 1979; Uchida *et al.*, 1981; Lange *et al.*, 1982; Jeppsson *et al.*, 1983; Dass & Bagshawe, 1984; Nustad *et al.*, 1984). However, the demonstration that smoking can produce false positive reactions (Tonik *et al.*, 1983; Maslow *et al.*, 1983) and the increasing evidence (Millán & Stigbrand, 1983; Paiva *et al.*, 1983) of the complexity of the genetic control of alkaline phosphatase expression, has made it difficult to interpret the results of these investigations.

A new approach to overcoming such problems has been the development of monoclonal antibodies (MAB) to PLAP. One of these, ICRF-H17E2, produced by initial immunization with term placental membranes (Travers & Bodmer, 1984; McLaughlin *et al.*, 1984), reacts with a heat resistant alkaline phosphatase that is more easily inhibitable by phenyl-alanyl-glycyl-glycine than L-leucine, thus, confirming that it recognises term PLAP. However, when compared with another anti-PLAP MAB (H317), H17E2 has additional reactivity with a L-leucine inhibitable PLAP-like alkaline phosphatase which can be extracted from normal testis (McLaughlin *et al.*, 1984). The monoclonal H17E2 also has been shown to react strongly with the surface membranes of all of a small number of germ cell tumours of the testis (Epenetos *et al.*, 1984).

It was therefore decided to use an assay based upon immunolocalisation of enzyme activity (ILEA), to evaluate the potential of H17E2 MAB serum assay for monitoring the treatment of patients with germ cell tumours of the testis. This paper reports the initial results of this study.

### Materials and methods

### *Serum samples*

These were selected from a bank of preserved (−20°C) specimens that had been collected since 1979 from patients with seminoma and other

*Present address: Department of Microbiology, Stanford University Medical School, Stanford, California, USA.
Correspondence: D.F. Tucker.
Received 22 August 1984; and in revised form 28 December 1984.

© The Macmillan Press Ltd., 1985

testicular germ cell tumours. The staging of disease in these patients and their treatment with conventional cis-platinum containing chemotherapy regimens, has been reported elsewhere (Oliver *et al.*, 1980, 1983*a*). The histological classification of these tumours was according to the system of the British Testicular Tumour Panel (Pugh & Cameron, 1976). All sera received for PLAP-like AP determinations had, in addition, been assayed for $\beta$-HCG, AFP and hydroxybutyric dehydrogenase (HBD). An assay for the latter enzyme was included with the established markers, because of its close relationship to lactic dehydrogenase which is known to be elevated in patients with testicular tumours. Control sera were from two sources: Red Cross donor samples remaining after routine Australia antigen screening and females from Guernsey in whom smoking history was known.

*Monoclonal antibody H17E2*

This murine IgG1 antibody was derived from a hybridoma produced after initial immunisation with purified plasma membranes of normal term placenta. It has been characterised (Travers & Bodmer, 1984) as reacting with all forms of heat stable PLAP, but not with the liver or intestinal isoenzymes of alkaline phosphatase.

*Immunolocalisation of enzyme activity (ILEA) assay*

Serum PLAP-like AP was determined in healthy individuals and patients with cancer, essentially as outlined in Figure 1. Optimal conditions for the assay were established by systematic variation of the coating antibody and test sample concentration and the various incubation times. Routinely, H17E2 MAB was adsorbed to plate wells by overnight incubation at 4°C with 100 $\mu$l (358 ng) of a Protein A affinity purified preparation (Hudson & Hay, 1980) in PBS. Unbound antibody was removed by washing with 0.05% Tween-20 in PBS, before addition of 100 $\mu$l aliquots of undiluted serum which were tested in duplicate. After incubation for 2 h at room temperature and further washing, the activity of the enzyme localised by the solid phase antibody was determined colorimetrically with Sigma 104 phosphatase substrate (100 $\mu$l per well of a 1 mg ml$^{-1}$ solution in 0.2 M diethanolamine containing 5 mM $MgCl_2$, pH 9.8). To develop the colour reaction, the plates, covered in aluminium foil, were incubated at 37°C for 2 h and then left overnight (16–17 h) at room temperature. For each assay, a calibration curve (Figure 2) was constructed with doubling dilutions of a normal term pregnancy serum. The heat stable PLAP activity (Sigma Units ml$^{-1}$) of the standard pregnancy serum was estimated by direct colorimetric determination (Sigma 15-min assay, Kit No. 104). A Sigma Unit is defined as that amount of enzyme activity that will liberate 1 $\mu$mol of *p*-nitrophenol per hour under the stated test conditions. Values expressed in Sigma Units ml$^{-1}$ were converted to International Units (iu l$^{-1}$) by multiplying by 16.7 as described in the assay kit instructions (Sigma technical bulletin No. 104).

**Figure 1** Determination of serum PLAP-like AP by immunolocalisation of enzyme activity (ILEA) assay.

**Figure 2** Variability associated with repeated ILEA assay of the same reference pregnancy serum.

### Results

### *Sensitivity and reproducibility of ILEA assay*

Each test run included a doubling dilution series of a reference near-term pregnancy serum made from stock aliquots (100 $\mu$l) of sera kept until use at $-70°C$. Figure 2 shows the results of 9 separate assays of the same pregnancy serum over a period of 6 months, with optical absorbance at 405 nm plotted on a log scale against enzymatic activity converted from Sigma Units ml$^{-1}$ to International Units (iu l$^{-1}$) (see **Methods**).

The variability at a fixed dilution is fairly constant on a logarithmic scale over the range and gives a coefficient of variation (C.V.) in the original units of $\pm$11.0%. However, it will be seen that there are substantial consistent differences between the 9 assays. It would thus be possible to eliminate part of this between-assay variability by using as standards for calibration, stored aliquots from a single dilution series of a large pregnancy serum pool. This would reduce the between-assay variability to a C.V. of $\pm$6.3%.

The background absorbance in these assays averaged 0.07 O.D. for wells ($n=61$) in which PBS was substituted for the test sera. Accordingly twice the background (0.14 O.D.) was considered to be the practical lower limit of detection of PLAP activity, an O.D. value corresponding to 0.04 iu l$^{-1}$ (Figure 2).

The results of selected sera from patients were similarly analysed to provide comparable estimates of inter-test variability both on single repeat testing (13 subjects) and serial sample testing during sustained remission (22 subjects; 2–7 samples each). For the blind re-testing figures the estimated C.V. of a single reading (the mean of 2 replicates) was $\pm$15.4%, whereas the repeated sample data gave a within-subject C.V. of $\pm$20.0%. Both these estimates of variability eliminate consistent differences between the results of successive assays, and so they should be compared with the reference serum assay variability of $\pm$6.3%. The larger C.V. of serial readings is most likely a result of genuine physiological changes within an individual from one time to another.

### *Serum PLAP-like AP levels in patients and controls*

Sera from two-hundred-and-thirteen Red Cross blood donor controls and from 50 patients with established metastatic testicular germ cell tumours were tested. The distribution of serum PLAP-like AP is shown in Figure 3. There were no significant differences between male and female control sera (Figure 3a), and the greater proportion (85% and 80% respectively) had assay readings in the range up to 0.2 O.D. The remaining group had a mean value of 0.36 O.D. in the case of the 24 male sera and 0.32 O.D. for the 11 female sera. The smoking habits of these 35 control individuals were ascertained, where possible, by postal questionnaire. This showed that 10 of the 24 male sera, and 7 of the 11 female sera with PLAP-like AP values >0.2 O.D., came from smokers. A further 3 male sera and 1 female serum were from ex-smokers but for the rest of the groups (11 males and 3 females) smoking details could not be obtained. Additional evidence for a smoking effect was obtained from a second control group, viz: 53 females from Guernsey, in whom smoking history was known (Figure 4). The data suggest a progressive mean rise in serum PLAP-like AP with increased smoking. This trend is highly significant ($P=0.0001$) as determined by a Wilcoxon rank regression test (Cuzick, in press).

In contrast, in the 50 patients with established metastatic disease (Figure 3*b*) serum values exceeding 0.2 O.D. were detected in 14 of the 16 with seminoma (88%), 7 of the 13 with mixed

**Figure 3** Distribution of serum PLAP-like AP levels in control blood donors (*a*) and pre-treatment groups of patients with metastatic testicular cancer (*b*). See calibration curve (**Figure 2**) for conversion of O.D. readings to iu $l^{-1}$.

**Figure 4** Effect of smoking on serum PLAP-like AP in a female control population. See calibration curve (**Figure 2**) for conversion of O.D. readings to iu $l^{-1}$.

**Figure 5** Individual serum PLAP-like AP values determined in patients (**Figure 3b**) with different metastatic tumour sub-types. See calibration curve (**Figure 2**) for conversion of O.D. readings to iu $l^{-1}$. (○) non-smoker; (●) smoker; (▲) ex-smoker.

seminoma and malignant teratoma (54%) and 7 of the 21 with malignant teratoma (33%). Details of the range of serum PLAP-like AP levels associated with each of these different tumour sub-types examined, are given in Figure 5. Again by Wilcoxon rank regression test, a significant ($P = 0.002$) trend was found for the frequency of elevated serum PLAP-like AP to be greater in seminoma than in mixed seminoma and malignant teratoma patients, and also in the latter group compared with the combined patients affected with various types of malignant teratoma.

*Effect of treatment on serum PLAP-like AP levels*

Table I shows several examples, in the different patient groups studied (Figure 3*b*, Figure 5), of serum PLAP-like AP determined before and after a successful course of treatment. Seven of these patients had moderate to marked (0.47–2.00 O.D.) elevations of PLAP-like AP before treatment, and a clear-cut (81–97%) reduction was apparent on achieving complete clearance of tumour. In the one patient who relapsed, a return to a positive reaction was detected by continued serial serum assay. Lesser (53–79%) reductions in PLAP-like AP could also be demonstrated in 10 of the remaining 15 patients. These decreases were smaller in percentage terms as the initial levels did not exceed 0.32 O.D. and the final values were only slightly less than the group above. Of the 5 patients with virtually unchanged serum PLAP-like AP, 4 were non-smokers and the other was an ex-smoker. A separate review of the smoking history of all patients in remission whose sera have been tested, established that the proportion of patients with sustained levels above 0.2 O.D. was greatest in smokers, intermediate in ex-smokers and lowest in non-smokers (29%, 17% and 2%, respectively).

**Table I** Changes in pre- and post-treatment serum PLAP-like AP according to tumour subtype and smoking status.

| | Serum PLAP-like AP (O.D. values)[a] | | |
|---|---|---|---|
| *Smoking status* | *Pre-treatment* | *Post-treatment* | *Disease status*[b] |
| *Seminomas* | | | |
| S | 2.0 | 0.06 | CR[c] |
| S | 1.4 | 0.24 | CR |
| Ex-S | 1.29 | 0.24 | CR |
| S | 0.47 | 0.06 | CR |
| S | 0.26 | 0.08 | 3 months |
| S | 0.25 | 0.10 | CR |
| Ex-S | 0.21 | 0.09 | CR |
| *Mixed tumours* | | | |
| Non-S | 1.5 | 0.09 | CR |
| S | 0.93 | 0.14 | CR |
| Non-S | 0.26 | 0.15 | CR |
| S | 0.20 | 0.09 | CR |
| Non-S | 0.21 | 0.09 | 4 months |
| Non-S | 0.14 | 0.06 | 2 months |
| Non-S | 0.09 | 0.08 | 10 months |
| Non-S | 0.11 | 0.09 | CR |
| *MTU* | | | |
| Non-S | 0.21 | 0.07 | CR |
| Non-S | 0.09 | 0.06 | CR |
| *MTI* | | | |
| Non-S | 0.13 | 0.06 | 1 month |
| S | 0.28 | 0.06 | 6 months |
| Ex-S | 0.17 | 0.09 | CR |
| *Unclassified* | | | |
| S | 0.79 | 0.13 | CR |
| S | 0.32 | 0.15 | CR |

[a]Optical absorbance at 405 nm. See calibration curve (**Figure 2**) for conversion of O.D. readings to iu $l^{-1}$.

[b]Assessed at 12 months unless otherwise stated.

[c]CR = Complete remission. No evidence of disease at 12 months.

*Correlation of PLAP-like AP expression with other tumour markers*

In addition to determining PLAP-like AP levels, pre-treatment sera from 32 of the patients included in this study, were also assayed for $\beta$-HCG, AFP and HBD. The results were analysed for a correlation of PLAP-like AP expression with these other tumour markers, using as the criteria for marker positivity, serum levels of >0.4 O.D. for PLAP-like AP, >10 ku $l^{-1}$ AFP, >1 $\mu g\, l^{-1}$ HCG and >200 iu $l^{-1}$ HBD. A raised serum PLAP-like AP was not found to be significantly associated (Yates corrected $\chi^2$ test) with either AFP, $\beta$-HCG or HBD, though, with one exception, all patients with elevated PLAP-like AP also had elevated HBD (Table II).

**Table II** Correlation between PLAP-like AP, alpha-foetoprotein, $\beta$-human chorionic gonadotrophin and hydroxybutyric dehydrogenase production in patients with testicular cancer.

| *Total N 32* | | *AFP* + | *AFP* − | *HCG* + | *HCG* − | *HBD* + | *HBD* − |
|---|---|---|---|---|---|---|---|
| PLAP- | + | 0 | 8 | 4 | 4 | 7 | 1 |
| LIKE AP | − | 9 | 15 | 11 | 13 | 12 | 12 |
| | | $\chi^2 = 2.52$ | | $\chi^2 = 0.04$ | | $\chi^2 = 2.12$ | |

### Discussion

Placental alkaline phosphatase was one of the first examples of the class of proteins now classified as oncofoetal antigens. The original report was from a patient with squamous cell carcinoma of the lung (Fishman *et al.*, 1968*a*), though subsequently similar aberrant expression was demonstrated in a small proportion of tumours from other sites such as bone marrow (Damle *et al.*, 1979), breast (Wada *et al.*, 1979), gut (Skinner & Whitehead, 1981) and gonad (Wahren *et al.*, 1979; Uchida *et al.*, 1981; Lange *et al.*, 1982; Haije *et al.*, 1979). The enzyme produced by the original patient's tumour (the Regan variant) was indistinguishable by catalytic and immunological criteria from the normal term placental enzyme (PLAP) (Fishman *et al.*, 1968*b*). However, as the phenomenon became more widely recognised, other PLAP variants (Nagao- and Kashara-type (Nakayama *et al.*, 1970; Higashino *et al.*, 1972)) were discovered. Subsequent detailed immunochemical studies of these variants suggested that they were either the products of other, less frequent, alleles of the PLAP locus (D for Nagao-type) (Inglis *et al.*, 1973) or were related to a form occurring in hepatoma patients and normal FL amnion cells (Kasahara-type (Higashino *et al.*, 1975)).

While it has been possible with polyclonal antisera to demonstrate that most seminomas express PLAP (Wahren *et al.*, 1979; Uchida *et al.*, 1981; Lange *et al.*, 1982; Jeppsson *et al.*, 1983), the identity of the particular variant involved remains uncertain. The results from a recent study of normal testis alkaline phosphatase using MABs to PLAP, suggest that the testis expresses a form of alkaline phosphatase which is controlled by a separate genetic locus (Millán & Stigbrand, 1983). Although this putative testis locus has different alleles to the PLAP locus, cross-reactions of testis type enzymes with some of the anti-PLAP monoclonal reagents can occur (Millán & Stigbrand, 1983). This enzyme in current parlance is described as testicular placental alkaline phosphatase-like alkaline phosphatase (testicular PLAP-like AP). Recent studies of the reactivity of the MAB H17E2 used for the present serum assays, have demonstrated that it recognizes both testicular PLAP-like AP and PLAP (Travers & Bodmer, 1984; McLaughlin *et al.*, 1984). Further preliminary analysis suggests that this antibody is monomorphic as it reacts with the products of all the frequent PLAP alleles (Harris, personal communication). However, its full pattern of reactivity with all the alleles of the testicular PLAP-like AP locus still remains to be established.

Despite these caveats, the data presented herein confirm earlier studies using polyclonal antibody-based assays that serum PLAP-like AP is markedly elevated in the majority of patients with metastatic seminoma, and returns to normal after successful treatment (Wahren *et al.*, 1979; Uchida *et al.*, 1981; Lange *et al.*, 1982; Jeppsson *et al.*, 1983; Dass & Bagshawe, 1984; Nustad *et al.*, 1984). Furthermore, in the present and two similar studies (Horwich *et al.*, 1985; Epenetos *et al.*, 1985) H17E2 immunoassay has detected positive reactivity in 88–100% of seminoma patients with active disease, a substantial improvement on the 50–71% positive rate observed with the aforementioned polyclonal antibody assays. In two previous reports (McLaughlin & Johnson, 1984; Millán *et al.*, 1982), H17E2 and F11 monoclonal immunoassay detected a lower incidence of, respectively, 4/9 and 3/6 seminoma patients with raised serum PLAP, although in both these investigations clinical details supporting the existence of metastases were lacking.

Two new observations also have been made in the course of the present serum marker evaluations. First, elevated levels in patients with malignant teratoma occurred more frequently if there was a mixed tumour with both seminoma and teratoma components. Thus, H17E2 MAB assay may also have potential in estimating the relative proportion of seminoma present in the metastases in these patients. The second observation relates to the failure to detect PLAP-like AP in sera of two patients, who had extensive metastatic malignant teratoma trophoblastic (MTT) and markedly elevated serum human $\beta$-HCG associated with their disease. It is possible that such patients' tumours are showing less than complete placental differentiation, or have failure of secretion of intracellular products. The latter explanation accords with the absence of raised PLAP-like AP in 18 sera (kindly supplied by Professor Bagshawe) from female patients with highly elevated $\beta$-HCG due to metastatic choriocarcinoma (Tucker, unpublished). A failure of secretion rather than expression is also consistent with the reported intracellular detection of a L-phenylalanine and L-leucine inhibitable form of PLAP by the choriocarcinoma line BEWO (Speeg *et al.*, 1977). Alternatively, the lack of circulating PLAP-like AP could be due to the tendency of MTT to express the products of early gestational trophoblast rather than those which appear predominantly after the first trimester. However, in one of these two MTT patients immunocytochemical staining of the tumour tissue demonstrated a high content of H17E2 reactive PLAP-like AP, even though the serum level of the enzyme was within normal limits (0.18 O.D.; Figure 5). Further study of this type of case may help clarify whether real differences do exist between the neoplastic expression and release of PLAP-like AP in seminoma and malignant teratoma.

Another clinically interesting finding is the one patient with metastatic seminoma who had serum AFP of 14,000 $ku\,l^{-1}$ and expression of AFP by his tumour as determined by immunocytochemical staining. Some investigators have considered the detection of AFP in the serum of patients with metastatic seminoma indicates the appearance of occult teratoma (Lange *et al.*, 1980). Others have regarded the appearance of this marker as supporting the idea that seminoma is an intermediate pathological state which evolves to either AFP- or HCG-secreting teratoma (Raghavan *et al.*, 1982). The PLAP-like AP level in the aforementioned seminoma patient with elevated AFP, was the lowest of all the patients with seminoma (0.06 O.D.; Figure 5). In addition, in the analysis of the patients with established metastases, there was a suggestion of a negative association of PLAP-like AP and AFP expression (Table II). These observations may be a reflection of an interaction between the genes controlling expression of these two cellular products, though study of a larger number of these rare AFP-producing seminomas is clearly necessary.

Previous reports (Tonik *et al.*, 1983; Maslow *et al.*, 1983) that smoking is an important contributory factor to raised levels of serum PLAP-like AP, have been substantiated by the present data obtained from patients in remission and both Guernsey and Red Cross Blood donor control populations. A history of smoking clearly limits the use of serum PLAP-like AP determinations to monitor patients with seminoma after treatment. However, serial assay in remission patients, even if they were smokers, was very reproducible so this should still provide a useful adjunct to radiological examinations for follow-up studies. Further investigations of the underlying causes of the increased levels in smokers will be required in order to improve the specificity of the assay and better understand the patho-physiological effects of smoking.

From a practical standpoint, it is important that the high specificity of the MAB H17E2 is exploitable in the ILEA assay with an inter-test reproducibility of PLAP-like AP measurements much the same as previously reported for polyclonal antibody ELISA (Millán & Stigbrand, 1981). These ELISA-type assays also compare favourably with radioimmunoassay, where within assay coefficients of variation for replicates of 3–8%, and between repeats of the same sample on different occasions of 8–20%, have been reported (Chard, 1982).

There are several potential advantages of the MAB based technique over radioimmunoassay, which should remain applicable with its adaptation to other tumour markers. In particular, ILEA assay does not require the special precautions and separation steps necessary for assays using radioactive material. The simplicity and sensitivity (down to 200 $ng\,l^{-1}$; P. Davis, personal communication) of the method together with easy automation of end-point reading should also make individual tests more cost effective. The ILEA assay clearly satisfies the main need for testis tumour marker monitoring of a simple, reliable and rapid technique available on site at the hospitals patients are attending for treatment. However, the economy possible with ILEA assay will be realized only where there is a greater demand for frequent serum PLAP-like AP assessment, such as exists in Regional Centres treating larger numbers of patients.

We are indebted to Professor A. Waters and the British Red Cross Blood Transfusion Service for enabling us to use residual sera for control determinations; Miss E. Hardwick of the Red Cross for help with obtaining information on smoking habits of blood donors; Professor Michael Healy and Dr Jack Cuzick for statistical analyses; Yong-Lan Pookim for expert technical assistance; Dr John Masters, Tissue Culture Laboratory, St Paul's Hospital, for providing assistance with storage and retrieval of sera; Dr A. Rose, St Paul's Hospital for performing HBD assays; and Professor T. Chard, St Bartholomew's Hospital, for performing AFP and HCG assays and Dr C. Parkinson for histological classification of the tumours. Our thanks are also due to Linda Hayes and Audrey Becket for assistance with preparation of the manuscript.

### References

CHARD, T. (1982). An introduction to radioimmunoassay and related techniques. Characteristics of binding assays-precision. In: *Laboratory Techniques in Biochemistry and Molecular Biology Vol. 6, Part 2.* (Eds. Work & Work), Amsterdam: Elsevier, p. 203.

CUZICK, J. (1985). A Wilcoxon-type test for trend. *Stat. Med.* (in press).

DAMLE, S.R., SHEETY, P.A., JUSSAWALLA, D.J., BHIDE, S.V. & BAXI, A.J. (1979). Occurrence of heat-labile Regan type alkaline phosphatase in hematopoietic tumors. *Int. J. Cancer*, **24**, 398.

DASS, S. & BAGSHAWE, K.D. (1984). A sensitive, specific radioimmunoassay for placental alkaline phosphatase. In: *Human Alkaline Phosphatases. Progress in Clinical and Biological Research, Vol. 166.* (Eds. Stigbrand & Fishman), New York: Alan R. Liss, Inc., p. 49.

EPENETOS, A.A., MUNRO, A.J., TUCKER, D.F. & 6 others. (1985). Monoclonal antibody assay of serum placental alkaline phosphatase in the monitoring of testicular tumours. *Br. J. Cancer*, **51** (this issue).

EPENETOS, A.A., TRAVERS, P., GATTER, K.C., OLIVER, R.T.D., MASON, D.Y. & BODMER, W.F. (1984). An immunohistological study of testicular germ cell tumours using two different monoclonal antibodies against placental alkaline phosphatase. *Br. J. Cancer*, **49**, 11.

FISHMAN, W.H., INGLIS, N.R., GREEN, S. & 6 others. (1968*a*). Immunology and biochemistry of Regan isoenzyme of alkaline phosphatase in human cancer. *Nature*, **219**, 698.

FISHMAN, W.H., INGLIS, N.R., STOLBACH, L.L. & KRANT, M.J. (1968*b*). A serum alkaline phosphatase isoenzyme of human neoplastic cell origin. *Cancer Res.*, **28**, 150.

HAIJE, W.G., MEERWALDT, J.H., TALERMAN, A. & 5 others. (1979). The value of sensitive assay of carcino-placental alkaline phosphatase (CPAP) in the follow-up of gynaecological cancers. *Int. J. Cancer*, **24**, 288.

HIGASHINO, K., HASHINOTSUME, M., YANG, K.Y., TAKAHASHI, Y. & YAMAMURA, Y. (1972). Studies on a variant alkaline phosphatase in sera of patients with hepatocellular carcinoma. *Clin. Chim. Acta*, **40**, 67.

HIGASHINO, K., KUDO, S., OHTANI, R., YAMAMURA, Y., HONDA, T. & SAKURAI, J. (1975). A hepatoma associated alkaline phosphatase, the Kasahara isoenzyme, compared with one of the isoenzymes of FL amnion cells. *Ann. N.Y. Acad. Sci.*, **259**, 337.

HORWICH, A., TUCKER, D.F. & PECKHAM, M.J. (1985). Placental alkaline phosphatase as a tumour marker in seminoma using the H17E2 monoclonal antibody assay. *Br. J. Cancer*, **51** (this issue).

HUDSON, L. & HAY, F.C. (1980). Isolation of IgG subclasses using protein A-sepharose. In: *Practical Immunology 2nd Edition*, Oxford: Blackwell Sci. Publ., p. 223.

INGLIS, N.R., KIRLEY, S., STOLBACH, L.L. & FISHMAN, W.H. (1973). Phenotypes of the Regan isoenzyme and identity between the placental D-variant and the Nagao enzyme. *Cancer Res.*, **33**, 1657.

JEPPSSON, A., WAHREN, B., STIGBRAND, T., EDSMYR, F. & ANDERSON, L. (1983). A clinical evaluation of serum placental alkaline phosphatase in seminoma patients. *Br. J. Urol.*, **55**, 73.

LANGE, P.H. (1982). Testicular cancer markers. In: *Human Cancer Markers, Vol. 2.* (Eds., Sell & Wahren), New Jersey: Humana Press, p. 259.

LANGE, P.H., MILLÁN, J.L., STIGBRAND, T., VESSELLA, R.L., RUOSLAHTI, E. & FISHMAN, W.H. (1982). Placental alkaline phosphatase as a tumor marker for seminoma. *Cancer Res.*, **42**, 3244.

LANGE, P.H., NOCHOMOVITZ, L.E., ROSAI, J. & 15 others. (1980). Serum alpha-fetoprotein and human chorionic gonadotrophin in patients with seminoma. *J. Urol.*, **124**, 472.

MASLOW, W.C., MUENSCH, H.A., AZAMA, F. & SCHNEIDER, A.S. (1983). Sensitive fluorometry of heat-stable alkaline phosphatase (Regan enzyme) activity in serum from smokers and non smokers. *Clin. Chem.*, **29**, 260.

McLAUGHLIN, P.J. & JOHNSON, P.M. (1984). A search for human placental-type alkaline phosphatases using monoclonal antibodies. In: *Human Alkaline Phosphatases. Progress in Clinical and Biological Research, Vol. 166.* (Eds. Stigbrand & Fishman), New York: Alan R. Liss, Inc., p. 67.

McLAUGHLIN, P.J., TRAVERS, P.J., McDICKEN, I.W. & JOHNSON, P.M. (1984). Demonstration of placental and placental-like alkaline phosphatase in non-malignant human tissue extracts using monoclonal antibodies in an enzyme immunoassay. *Clin. Chim. Acta*, **137**, 341.

MILLÁN, J.L. & STIGBRAND, T. (1981). "Sandwich" enzyme immunoassay for placental alkaline phosphatase. *Clin. Chem.*, **27**, 2014.

MILLÁN, J.L. & STIGBRAND, T. (1983). Antigenic determinants of human placental and testicular placental-like alkaline phosphatases as mapped by monocloncal antibodies. *Eur. J. Biochem.*, **136**, 1.

MILLÁN, J.L., STIGBRAND, T., RUOSLAHTI, E. & FISHMAN, W.H. (1982). Characterization and use of an allotype-specific monoclonal antibody to placental alkaline phosphatase in the study of cancer-related phosphatase polymorphism. *Cancer Res.*, **42**, 2444.

NAKAYAMA, T., YOSHIDA, M. & KITAMURA, M. (1970). L-leucine-sensitive, heat-stable alkaline phosphatase isoenzyme detected in a patient with pleuritis carcinomatosa. *Clin. Chim. Acta*, **30**, 546.

NEWLANDS, E.S., RUSTIN, G.J.S., BEGENT, R.H.J., PARKER, D. & BAGSHAWE, K.D. (1983). Further advances in the management of malignant teratomas of the testis and other sites. *Lancet*, **i**, 948.

NUSTAD, K., MONRAD-HANSEN, H.P., PAUS, E., MILLÁN, J.L., NØRGAARD-PEDERSEN & THE DATECA STUDY GROUP. (1984). Evaluation of a new, sensitive radio-immunoassay for placental alkaline phosphatase in pre- and post-operative sera from the Danish testicular cancer material. In: *Human Alkaline Phosphatases Progress in Clinical and Biological Research, Vol. 166.* (Eds. Stigbrand & Fishman), New York: Alan R. Liss, Inc., p. 337.

OLIVER, R.T.D., BLANDY, J.P., HENDRY, W.F., PRYOR, J.P., WILLIAMS, J.P. & HOPE-STONE, H.F. (1983*a*). Evaluation of radiotherapy and/or surgico-pathological staging after chemotherapy in the management of metastatic germ cell tumours, *Br. J. Urol.*, **55**, 764.

OLIVER, R.T.D., HOPE-STONE, H.F. & BLANDY, J.P. (1983*b*). Justification of the use of surveillance in the management of stage I germ cell tumours of the testis. *Br. J. Urol.*, **55**, 760.

OLIVER, R.T.D., ROHATINER, A., WRIGLEY, P.F.M. & MALPAS, J.S. (1980). Chemotherapy of metastatic testicular tumours. *Br. J. Urol.*, **52**, 34.

PAIVA, J., DAMJANOV, I., LANGE, P.H. & HARRIS, H. (1983). Immunohistochemical localization of placental-like alkaline phosphatase in testis and germ cell tumors using monoclonal antibodies. *Am. J. Pathol.*, **111**, 156.

PECKHAM, M.J., BARRETT, A., HORWICH, A. & HENDRY, W.F. (1983). Orchiectomy alone for stage I testicular non-seminoma. A progress report. *Br. J. Urol.*, **55**, 754.

PUGH, R.C.B. & CAMERON, K.M. (1976). Teratoma. In: *Pathology of the Testis.* (Ed. Pugh), Oxford: Blackwell Sci. Publ., p. 199.

RAGHAVAN, D., GIBBS, J., NOGUEIRA, C.R. & 4 others. (1980). The interpretation of marker protein assays: a critical appraisal in clinical studies and a zenograft model. *Br. J. Cancer*, **41** (Suppl. IV), 191.

RAGHAVAN, D., SULLIVAN, A.L., PECKHAM, M.J. & NEVILLE, A.M. (1982). Elevated serum alphafetoprotein and seminoma: clinical evidence for a histologic continuum? *Cancer*, **50**, 982.

SKINNER, J.M. & WHITEHEAD, R. (1981). Carcinoplacental alkaline phosphatase in malignant and premalignant conditions of the human digestive tract. *Virchows Archiv* [A], **394**, 109.

SPEEG, K.V., AZIZKHAN, J.C. & STROMBERG, K. (1977). Characteristics of alkaline phosphatase from two continuous lines of human choriocarcinoma cells. *Exp. Cell Res.*, **105**, 199.

TONIK, S.E., ORTEMEYER, A.E., SHINDELMAN, J.E. & SUSSMAN, H.H. (1983). Elevation of serum placental alkaline phosphatase levels in cigarette smokers. *Int. J. Cancer*, **31**, 51.

TRAVERS, P. & BODMER, W. (1984). Preparation and characterisation of monoclonal antibodies against placental alkaline phosphatase and other human trophoblast-associated determinants. *Int. J. Cancer*, **33**, 633.

UCHIDA, T., SHIMODA, T., MIYATA, H. & 6 others. (1981). Immunoperoxidase study of alkaline phosphatase in testicular tumors. *Cancer*, **48**, 1455.

WADA, G.H., SHINDELMAN, J.E., ORTEMEYER, A.E. & SUSSMAN, H.H. (1979). Demonstration of placental alkaline phosphatase in human breast cancer. *Int. J. Cancer*, **23**, 781.

WAHREN, B., HOLMGREN, P.A. & STIGBRAND, T. (1979). Placental alkaline phosphatase, alphafetoprotein and carcinoembryonic antigen in testicular tumours. Tissue typing by means of cytologic smears. *Int. J. Cancer*, **24**, 749.